# A Case of a Splitting Headache: Paraneoplastic Rhombencephalitis

**DOI:** 10.7759/cureus.24302

**Published:** 2022-04-20

**Authors:** Nico Gotera, Christian P Schultheis

**Affiliations:** 1 Internal Medicine Residency, MercyOne North Iowa Medical Center, Mason City, USA; 2 Hematology and Medical Oncology, Mission Cancer + Blood, Des Moines, USA

**Keywords:** paraneoplastic neurologic syndrome, acetylcholine ganglionic receptor antibody, paraneoplastic encephalomyelitis, small-cell lung carcinoma, rhomboencephalitis, paraneoplastic rhomboencephalitis

## Abstract

Paraneoplastic neurologic syndromes are a set of rare neurological conditions with a wide variety of presentations, ranging from headache to gait imbalance. These conditions are often underreported and underdiagnosed. Paraneoplastic rhombencephalitis is a subtype that involves inflammation of the hindbrain. This case involves a 67-year-old female with metastatic small-cell lung cancer who acutely developed neurological symptoms with magnetic resonance imaging findings consistent with rhombencephalitis. Our case discusses the updated diagnostic criteria for paraneoplastic neurologic syndrome released in July 2021 compared with the prior criteria in 2004. In addition, it illustrates the importance of increasing awareness of this condition for early diagnosis and prompt treatment, which can potentially influence morbidity outcomes.

## Introduction

Paraneoplastic neurologic syndromes (PNS) are a constellation of syndromes associated with varying presentations of neurological symptoms in the setting of malignancy [1]. Overall, PNS occurs in about one out of three hundred patients, with notably few epidemiologic studies reported [2,3]. The reported incidence of PNS, as related to all tumors, has a wide variation from 1.6 to 8.9 per million person-years, which suggests that PNS remains underrecognized and underreported [2,3]. One of the classic presentations is paraneoplastic encephalomyelitis, which is neural dysfunction involving at least two or more regions of the neurological system, including multiple areas of the cerebrum, brainstem, cerebellum, spinal cord, and/or autonomic nervous system [1]. The most common underlying malignancy involved in PNS is small-cell lung cancer, which is found in 75% of cases. Paraneoplastic rhombencephalitis is a subset of paraneoplastic encephalomyelitis, which involves inflammation of the hindbrain, specifically the brainstem and cerebellum [1]. Here, we present the case of a 67-year-old female with a medical history of metastatic small-cell lung cancer who acutely developed paraneoplastic rhombencephalitis with prior stable disease.

## Case presentation

**Figure 1:**
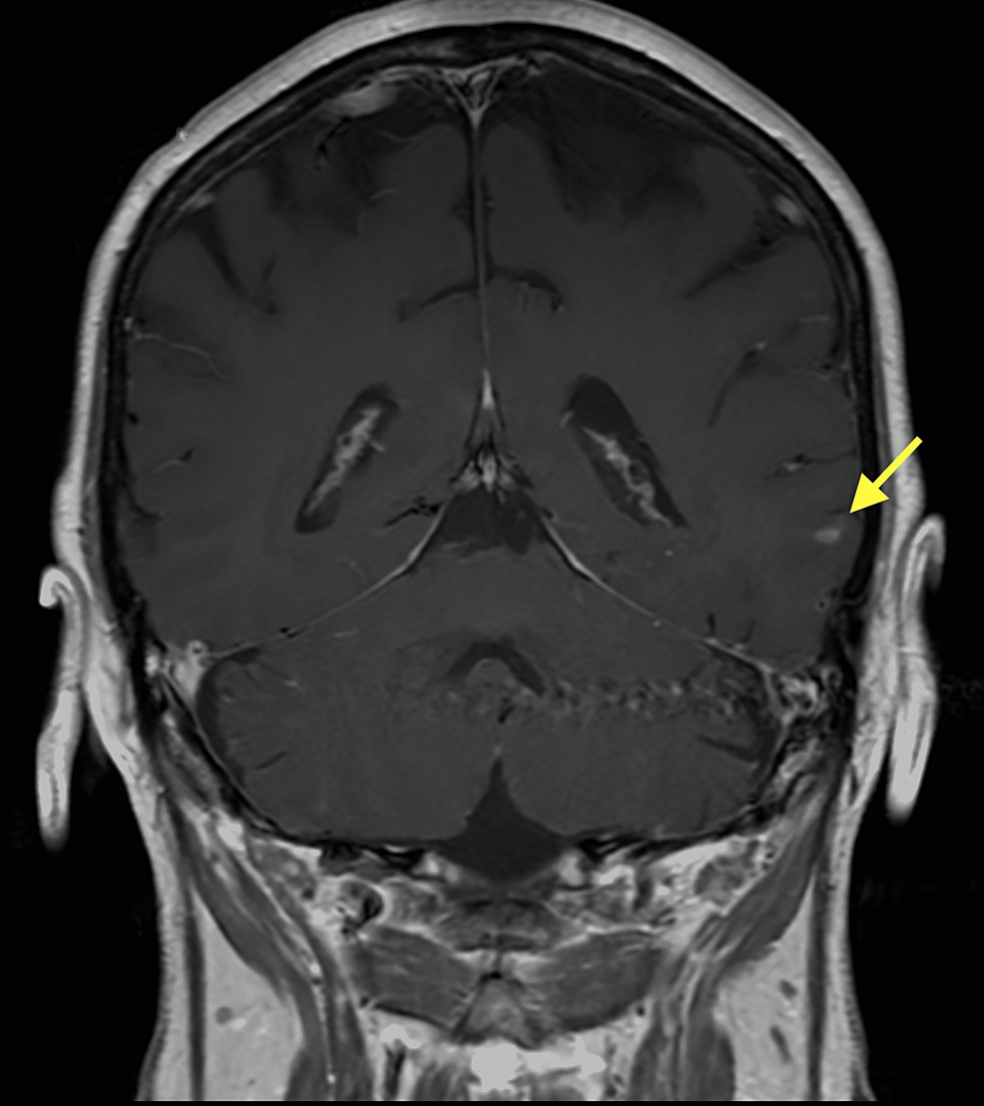
Brain MRI coronal T1 post-contrast image 12 days prior to admission. The yellow arrow shows stable enhancing focus lateral left temporal lobe from previously treated metastatic disease. MRI: magnetic resonance imaging

**Figure 2:**
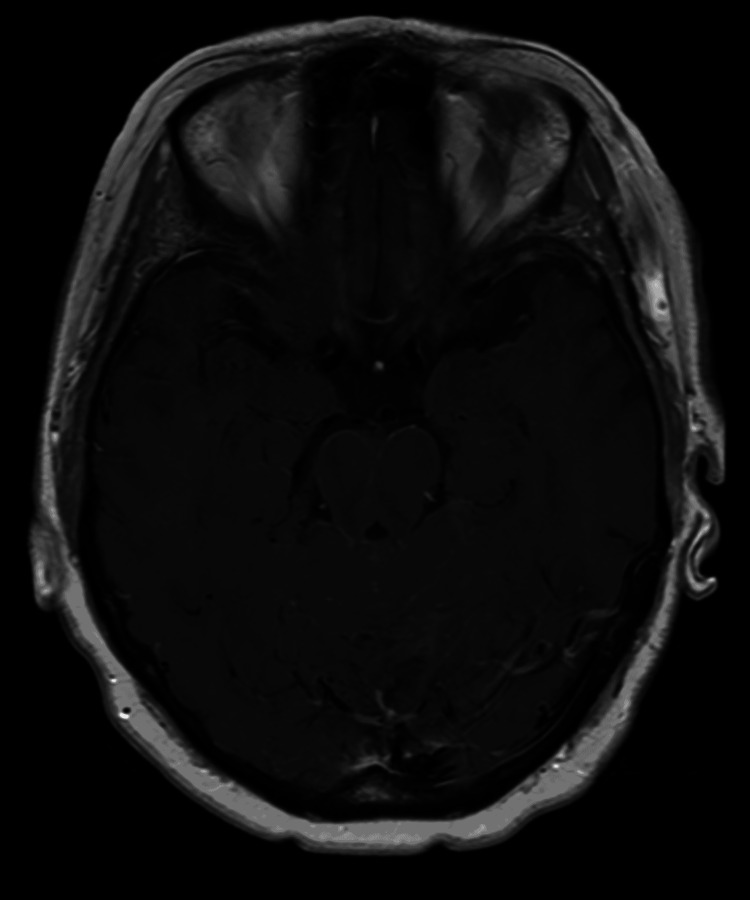
Brain MRI axial T1 post-contrast 12 days prior to admission. MRI: magnetic resonance imaging

A 67-year-old female with a medical history relevant for metastatic small-cell lung cancer to the liver and multiple bones presented with sudden-onset ataxia and splitting headache for one week to the hematology-oncology clinic. There were no known triggering factors. She had associated symptoms of lightheadedness, slurred speech, nausea, vomiting, and splitting headache over the course of one week. Two weeks prior to presentation, the patient underwent magnetic resonance imaging (MRI) of the brain during routine follow-up that did not show any metastatic disease (Figures 1, 2).

For her medical history, she was originally diagnosed with small-cell lung cancer a year and a half prior to presentation after a lung cancer screening computed tomography (CT) showed a left lower lobe spiculate mass and left hilar and mediastinal adenopathy. Staging positron emission tomography-computed tomography (PET-CT) showed multiple bony metastases including the T6 vertebral body, right periacetabular ischium, left sacral ala, and the proximal left humeral metaphysis. CT-guided biopsy of her right ischium lesion showed metastatic small-cell carcinoma. Initial MRI of the brain for staging was negative for metastatic disease. This initial staging for the patient confirmed her to have an extensive-stage disease. Prophylactic central nervous system (CNS) radiation was deferred at the time of initial evaluation through collaborative decision-making with the patient. She received atezolizumab, carboplatin, and etoposide chemotherapy for four cycles, and a follow-up CT of the lungs showed a mixed mediastinal and pulmonary response, with mild improvement on the right and probable mild progression on the left. She was then placed on maintenance atezolizumab for 17 cycles lasting about nine months before it was discontinued due to the development of grade 3-4 pneumonitis from her immunotherapy. Surveillance MRI of the brain around the end of this period showed a solitary brain lesion, which was later treated with stereotactic radiosurgery via cyberknife intervention. A follow-up of the brain one month later showed the lesion increased in size after cyberknife. Follow-up three months later, the lesion was shown to be decreasing in size, indicating a partial response. She later developed coronavirus disease 2019 pneumonia and was admitted. She was adequately treated with steroids and remdesivir and discharged on 6 L of oxygen. She was eventually able to tolerate being on room air during the day and on 2 L of oxygen at night. Follow-up chest CT three months later showed new hypodense liver lesions, which were later biopsied showing metastatic small-cell lung cancer. She was then started on carboplatin and etoposide for about six cycles lasting approximately three months. Atezolizumab was not continued due to side effects of pneumonitis. During that period, she later developed acute shortness of breath and was noted to be hypoxic around the mid-80s on room air. She was later found to have a right-sided submassive pulmonary embolism, which was treated with anticoagulation. She was able to be discharged on room air. She had follow-up imaging with CT chest, abdomen, and pelvis showing partial response to the palliative chemotherapy and no new evidence of metastatic disease. Her most recent MRIs on follow-up showed stable disease (Figures 1, 2). On follow-up visits, the patient had otherwise been doing well throughout the course of palliative chemotherapy. Her Eastern Cooperative Oncology Group (ECOG) performance status at this time was two, which is defined as being ambulatory and capable of all self-care but not able to carry out work activities [4]. Patients are also up and able for more than 50% of their waking hours [4].

**Figure 3:**
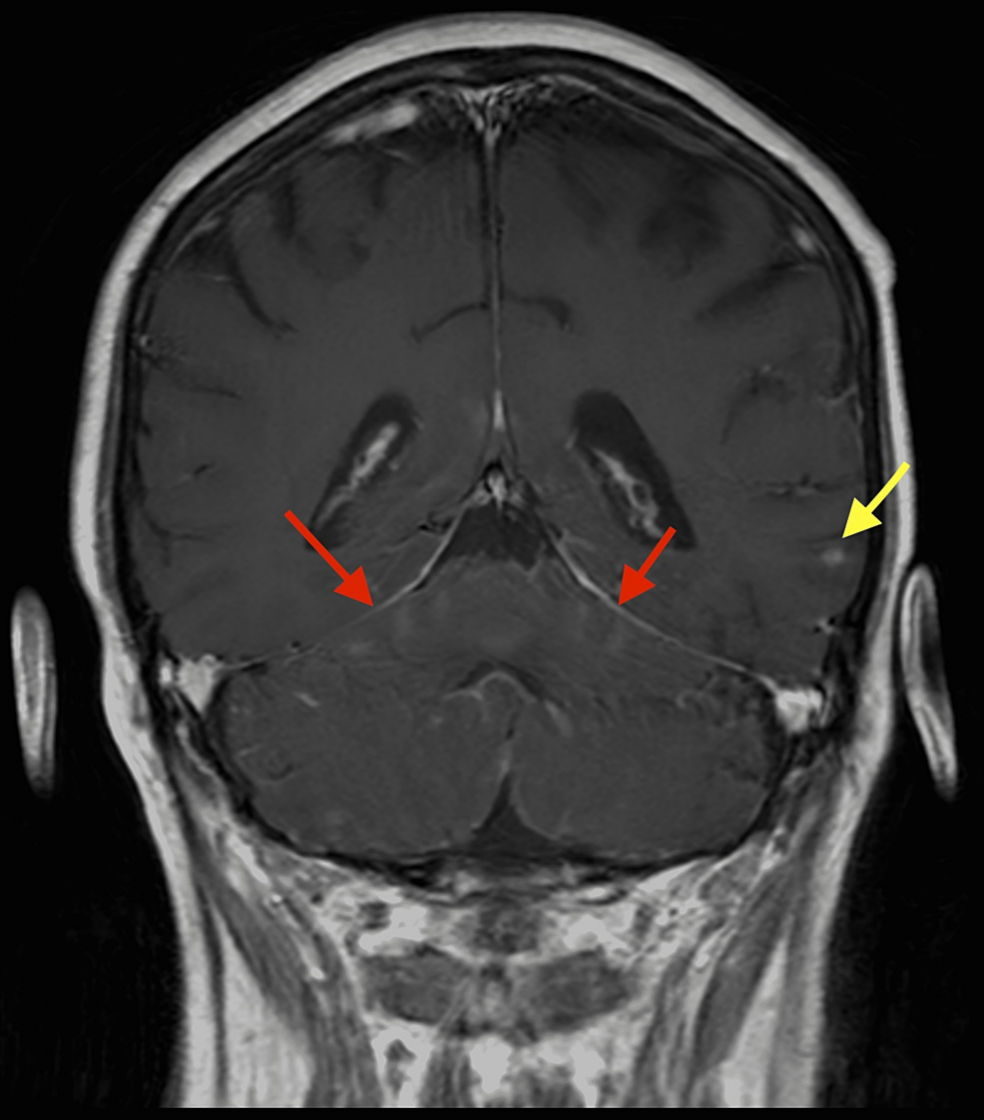
Brain MRI coronal view during T1 post-contrast showing interval leptomeningeal enhancement (red arrow) during hospitalization. The yellow arrow shows stable enhancing focus lateral left temporal lobe from previously treated metastatic disease. MRI: magnetic resonance imaging

**Figure 4:**
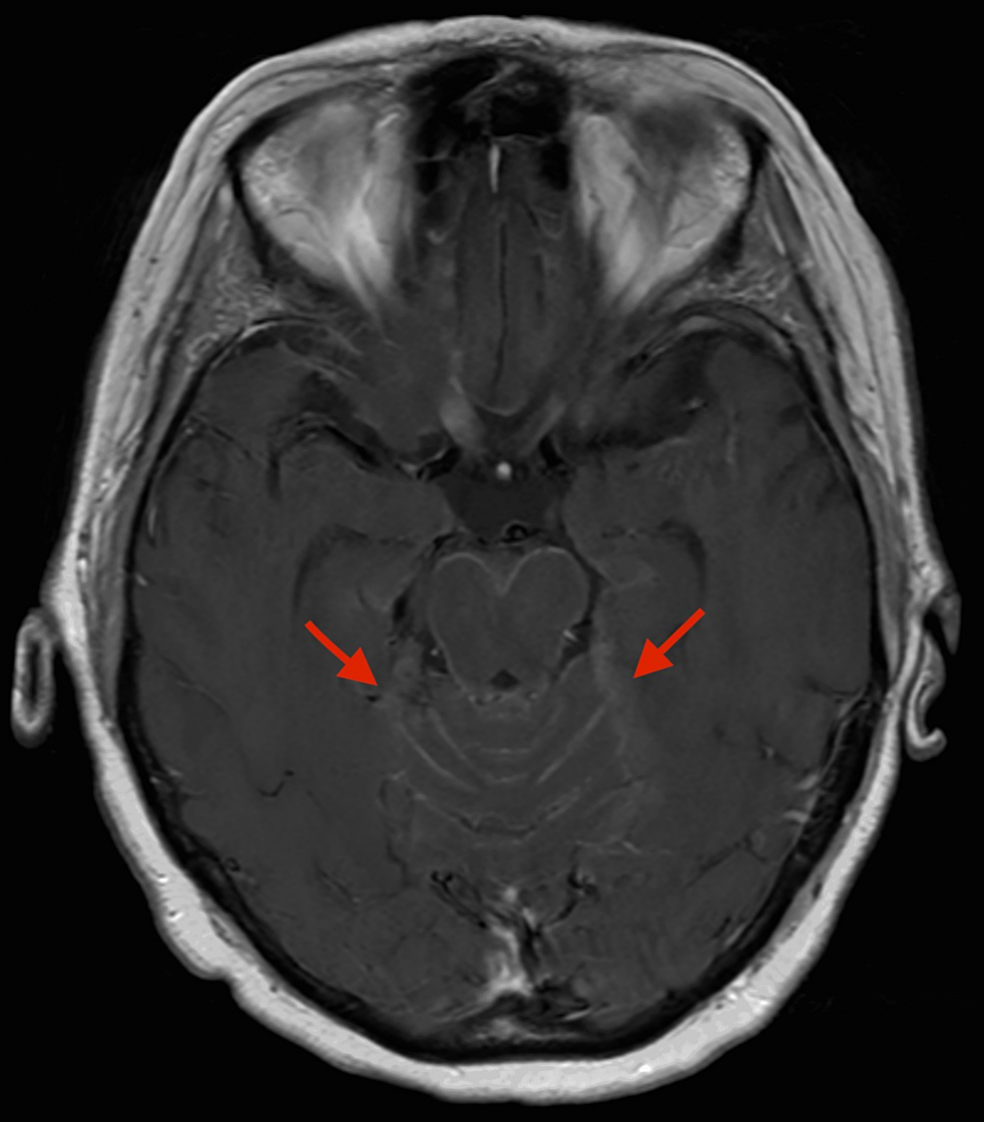
Brain MRI axial view during T1 post-contrast showing interval increase in leptomeningeal enhancement (red arrows) during admission. MRI: magnetic resonance imaging

Upon presentation to our clinic, she was afebrile and did not have any vital sign irregularities. On physical examination, she was slurring her words but did not appear to have any focal neuro deficits on neuro examination, including cranial nerves II-XII intact, strength 5/5 in upper and lower extremities, and no sensory deficits. However, her gait was not formally assessed. She was directly admitted to the hospital and started on supportive care with antiemetics and fluids. Her complete blood cell count, complete metabolic panel, urinalysis, and chest X-ray were all unremarkable. A repeat MRI of the brain showed leptomeningeal enhancement predominantly involving the posterior structures consistent with rhombencephalitis. She had a stable 3 mm enhancing focus in the lateral left temporal lobe from the previously treated metastatic disease from the cyberknife (Figures 3, 4). A lumbar puncture was performed after her MRI of the brain which showed glucose of 24 mg/dL, protein of 233.7 mg/dL, and neutrophil count of 25 cells/µL. Normal levels in our lab for protein CSF are 15-45 mg/dL. A paraneoplastic panel of her cerebrospinal fluid (CSF) and serum was remarkable for positive serum AChR ganglionic neuronal antibody (0.07 nmol/L). The normal range at our facility is less than 0.02 nmol/L. The rest of her serum and CSF panel was negative for anti-neuronal nuclear antibody, Purkinje cell antibody, collapsin response-mediator protein-5, anti-Gail nuclear antibody, amphiphysin antibody, anti-neuronal nuclear antibody, P/Q-type calcium channel antibody, and neuronal (V-G) K+ channel antibody. After receiving the paraneoplastic panel results, the patient was planned to be started on intravenous immunoglobulin (IVIG) 1 g/kg for two days and methylprednisolone 1,000 mg daily for five days. However, after completing IVIG treatment on the first day, the patient developed status epilepticus with minimal response to antileptics and was intubated. Due to the poor prognosis of the patient, her family decided to place the patient on comfort care and she later passed away.

## Discussion

In July 2021, the Paraneoplastic Neurologic Syndrome Care (PNS-Care) Panel published new guidelines on diagnosing PNS to update the previously recommended diagnostic criteria back in 2004 with recent advances in antibodies and clinical presentations in PNS [5]. Previously, the definition of PNS involved classical versus nonclassical syndromes. Classical syndromes included encephalomyelitis, limbic encephalitis, subacute cerebellar degeneration, sensory neuronopathy, opsoclonus-myoclonus, Lambert-Eaton myasthenic syndrome, chronic obstructive gastrointestinal pseudoobstruction, and dermatomyositis [6]. The presentation of a classical syndrome with a cancer diagnosis that had developed within five years of neurological symptoms was sufficient to diagnose PNS, such as in the case of our patient. If the patient had a nonclassical syndrome, then a well-characterized onconeuronal antibody, such as anti-Hu, Yo, CV2, Ri, MA2, or amphiphysin, with the diagnosis of cancer within five years of development of neurological symptoms was also sufficient for diagnosis. However, due to the findings of new antibodies and the challenges of false positives and discordant antibody tests and malignancy associations, the most recent published guidelines stepped away from these definitions to better define the definition of PNS [5]. The PNS-Care Panel created a scoring system known as the PNS-Care Score based on the following factors of the clinical level also known as phenotype or presentation, levels of antibody risk, and the finding of malignancy. The previously described classical syndromes are now known as high-risk phenotypes while the well-characterized onconeuronal antibodies are now described as high-risk antibodies [5]. Intermediate-risk phenotypes and antibodies include other neurological presentations and antibodies that are found to have some level of association with malignancy. Overall, the goals of these guidelines were to have a stronger requirement of neuronal antibodies with malignancy and move away from the five-year requirement of a cancer diagnosis. It is worth noting that according to the previous 2004 definition of PNS our patient had definite PNS. However, based on the most recently released guidelines, our patient was scored as probable PNS based on the high-risk phenotype, known malignancy history, but lack of known antibody associated with malignancy.

In addition, our patient was positive for acetylcholine ganglionic receptor antibody, which was not described in the most recent guidelines as a high or intermediate-risk antibody. One case-control study involving 15,000 patients at Mayo Clinic who were evaluated for paraneoplastic neurologic autoimmunity had approximately 1% of the patients positive for acetylcholine ganglionic receptor antibody. Of the half of those who were antibody-positive and evaluated for malignancy, 30% were found to have a general underlying malignancy [7]. Levels greater than 1.0 nmol/L were associated with autoimmune etiologies, malignancies, and paraneoplastic phenomena. Lower levels of acetylcholine ganglionic receptor antibodies had more diverse etiologies including peripheral neuropathy and CNS disorders. Those who had associated malignancy typically had small-cell lung cancer or thymoma. Their conclusion was that acetylcholine ganglionic receptor antibody aids in the diagnosis of malignancy [7]. While our patient had a low level of acetylcholine ganglionic receptor antibody, it fit in the context of our patient having small-cell lung cancer [7]. Based on the recent July 2021 guidelines, they recommended retesting serum and CSF samples for high and intermediate-risk antibodies, especially if clinical suspicion is high [5]. Furthermore, retesting should also occur if the antibody is unexpectedly positive for a malignancy that is typically not associated with it [5]. Retesting did not occur in our patient’s case, especially because the recommendation came out recently in the setting of this patient’s presentation. Future approaches for PNS should take the approach of retesting, especially if there is a high degree of suspicion for PNS.

Regarding our patient’s diagnosis of rhombencephalitis, the different possible etiologies for this included infectious, autoimmune, and paraneoplastic [8]. Our patient had no evidence of infection based on lack of fever, normal white blood cell count, and negative findings of enterovirus and herpesvirus on lumbar puncture. Furthermore, the most common cause of infection-related rhombencephalitis is associated with listeria, which often has an abscess with ring enhancement in the brain. Our patient lacked this finding. For autoimmune-related causes such as systemic lupus erythematosus and relapsing polychondritis, our patient had no other symptoms to suggest this. In addition, the acute change in neurological symptoms points against this. This ultimately leaves paraneoplastic syndrome as the most probable cause given the patient’s known malignancy history and high-risk phenotype. Furthermore, the most common cause of paraneoplastic rhombencephalitis is small-cell lung cancer, which is consistent with our patient’s diagnosis.

Overall, it has been reported that patients with paraneoplastic rhomboencephalitis tend to have high morbidity [9,10]. One review article suggests that morbidity is influenced by neurologic dysfunction rather than the underlying malignancy itself [11]. Treatment is based on treating the underlying malignancy, immunosuppression with antibody direct therapies, or T-cell-directed therapies in addition to treating symptoms [1]. However, it has been described that antibody-directed therapies, such as IVIG, are ineffective in some patients, which could have been a factor in the outcome in our patient’s case [1,12,13]. Some case reports suggest that T-cell-directed therapies have also been effective [13]. However, removing the tumor along with immunotherapy is associated with full recovery and decreased likelihood of the patient having a relapse in symptoms. In addition, early and aggressive treatment can ultimately influence the outcome in a patient [14]. This is also complicated by the use of immunosuppression, which can be counterintuitive to treating the malignancy with immunotherapy [12]. The patient had been otherwise stable based on CT imaging prior to admission. The earlier intervention of her paraneoplastic rhomboencephalitis could have potentially influenced her morbidity outcome by improving her neurological symptoms and prolonging survival [14]. Overall, it is important to treat paraneoplastic rhomboencephalitis early even without a definitive diagnosis because it can influence outcomes.

## Conclusions

Paraneoplastic rhombencephalitis is a rare and underdiagnosed condition under the subset of PNS. For oncological patients presenting with new-onset neurologic phenomena, it is important to consider this condition and other PNS early in the diagnosis, especially because its mortality is high. This requires a prompt MRI of the brain and lumbar puncture as well as a serum and CSF paraneoplastic panel. In addition, diagnosing PNS according to the recent July 2021 PNS-Care Panel guidelines suggests obtaining antibody measurements specific to the underlying malignancy to prevent false positives. The panel also recommended retesting patients for antibodies when there are any unexpected antibody results, such as negative antibodies with high clinical suspicion for PNS or positive antibodies with an unexpected underlying malignancy. Furthermore, if there is a high suspicion of PNS, it is important to perform early treatment interventions even without confirmation of PNS because of the high mortality risk. For our patient, it would have been important to retest antibodies given her high clinical suspicion for the paraneoplastic syndrome, as well as start her treatment immediately without waiting for results to return. Early recognition of PNS can affect morbidity for previously stable oncology patients, especially once future interventions are better understood.

## References

[1] Tirthani E, Said MS, Smith RG (2022). Paraneoplastic encephalomyelitis. StatPearls [Internet].

[2] Vogrig A, Gigli GL, Segatti S (2020). Epidemiology of paraneoplastic neurological syndromes: a population-based study. J Neurol.

[3] Hébert J, Riche B, Vogrig A (2020). Epidemiology of paraneoplastic neurologic syndromes and autoimmune encephalitides in France. Neurol Neuroimmunol Neuroinflamm.

[4] Azam F, Latif MF, Farooq A, Tirmazy SH, AlShahrani S, Bashir S, Bukhari N (2019). Performance status assessment by using ECOG (Eastern Cooperative Oncology Group) score for cancer patients by oncology healthcare professionals. Case Rep Oncol.

[5] Graus F, Vogrig A, Muñiz-Castrillo S (2021). Updated diagnostic criteria for paraneoplastic neurologic syndromes. Neurol Neuroimmunol Neuroinflamm.

[6] Graus F, Delattre JY, Antoine JC (2004). Recommended diagnostic criteria for paraneoplastic neurological syndromes. J Neurol Neurosurg Psychiatry.

[7] McKeon A, Lennon VA, Lachance DH, Fealey RD, Pittock SJ (2009). Ganglionic acetylcholine receptor autoantibody: oncological, neurological, and serological accompaniments. Arch Neurol.

[8] Campos LG, Trindade RA, Faistauer Â, Pérez JA, Vedolin LM, Duarte JÁ (2016). Rhombencephalitis: pictorial essay. Radiol Bras.

[9] Rees JH (2004). Paraneoplastic syndromes: when to suspect, how to confirm, and how to manage. J Neurol Neurosurg Psychiatry.

[10] Pelosof LC, Gerber DE (2010). Paraneoplastic syndromes: an approach to diagnosis and treatment. Mayo Clin Proc.

[11] Devine MF, Kothapalli N, Elkhooly M, Dubey D (2021). Paraneoplastic neurological syndromes: clinical presentations and management. Ther Adv Neurol Disord.

[12] Grisold W, Giometto B, Vitaliani R, Oberndorfer S (2011). Current approaches to the treatment of paraneoplastic encephalitis. Ther Adv Neurol Disord.

[13] Loehrer PA, Timmermann L, Pehl A, Bien CI, Pfestroff A, Pedrosa DJ (2020). Rhombencephalitis associated with isolated Zic4-antibodies in paraneoplastic cerebellar degeneration: a case report. BMC Neurol.

[14] Höftberger R, Rosenfeld MR, Dalmau J (2015). Update on neurological paraneoplastic syndromes. Curr Opin Oncol.

